# Epidermal RORα Maintains Barrier Integrity and Prevents Allergic Inflammation by Regulating Late Differentiation and Lipid Metabolism

**DOI:** 10.3390/ijms251910698

**Published:** 2024-10-04

**Authors:** Xiangmei Hua, Maria K. Ficaro, Nicole L. Wallace, Jun Dai

**Affiliations:** 1 School of Pharmacy, The University of Wisconsin, Madison, WI 53705, USA; xhua23@wisc.edu (X.H.); mficaro@wisc.edu (M.K.F.); nicolewallace2023@u.northwestern.edu (N.L.W.); 2 Carbone Cancer Center, The University of Wisconsin, Madison, WI 53705, USA; 3 Skin Disease Research Center, The University of Wisconsin, Madison, WI 53705, USA

**Keywords:** RORalpha, epidermal barrier, keratinocyte differentiation, lipid metabolism, contact dermatitis

## Abstract

The skin epidermis provides a barrier that is imperative for preventing transepidermal water loss (TEWL) and protecting against environmental stimuli. The underlying molecular mechanisms for regulating barrier functions and sustaining its integrity remain unclear. RORα is a nuclear receptor highly expressed in the epidermis of normal skin. Clinical studies showed that the epidermal RORα expression is significantly reduced in the lesions of multiple inflammatory skin diseases. In this study, we investigate the central roles of RORα in stabilizing skin barrier function using mice with an epidermis-specific *Rora* gene deletion (*Rora^EKO^*). While lacking spontaneous skin lesions or dermatitis, *Rora^EKO^* mice exhibited an elevated TEWL rate and skin characteristics of barrier dysfunction. Immunostaining and Western blot analysis revealed low levels of cornified envelope proteins in the *Rora^EKO^* epidermis, suggesting disturbed late epidermal differentiation. In addition, an RNA-seq analysis showed the altered expression of genes related to “keratinization” and “lipid metabolism” in RORα deficient epidermis. A lipidomic analysis further uncovered an aberrant ceramide composition in the *Rora^EKO^* epidermis. Importantly, epidermal *Rora* ablation greatly exaggerated percutaneous allergic inflammatory responses to oxazolone in an allergic contact dermatitis (ACD) mouse model. Our results substantiate the essence of epidermal RORα in maintaining late keratinocyte differentiation and normal barrier function while suppressing cutaneous inflammation.

## 1. Introduction

The skin epidermis is a vital barrier that averts water loss, maintains body temperatures, and shields against external assaults [1,2]. The disruption of skin barrier functions leads to diseases ranging from ichthyosis, a dry and scaly skin phenotype, to allergic diseases such as atopic dermatitis (AD) [3,4]. Therefore, establishing regulatory mechanisms for barrier integrity and maintenance is paramount for developing de novo therapeutics to combat pathological skin disorders.

Epidermal barrier function is primarily achieved at the outmost layer of the epidermis, called the stratum corneum (SC), composed of anuclear dead keratinocytes (corneocytes) and the surrounding lipid matrix. As the final product of keratinocyte terminal differentiation, each corneocyte contains a cornified envelope (CE) made up of cross-linked proteins and an externally linked lipid layer called the cornified lipid envelope (CLE) [5,6]. Appropriate protein and lipid components in the SC are acquired during the progression of keratinocyte differentiation. While the intermediate filaments keratin 5/14 (K5/14) and K1/10 are expressed in the proliferative basal layer and early differentiating spinous layers, respectively, key proteins with cross-linking activities, such as loricrin and filaggrin, are expressed in the granular layers during late differentiation [7]. At this stage, keratinocytes also produce unique skin lipids, including ultralong-chain (ULC) ω-acylceramides (CER_EOS), which are the precursors of ceramides bound to involucrin and loricrin in the CLE [1,8,9].

Normal skin barrier functions rely on a proper composition of proteins and lipids. Genetic studies show that loss-of-function mutations of *FLG*, the gene coding profilaggrin, are the primary cause of ichthyosis vulgaris and the predisposition factor for atopic dermatitis (AD) and other allergic diseases such as asthma and food allergies [10,11]. Recent lipidomic studies uncovered aberrant lipid profiles in AD skin lesions, such as a shift from long-chain to short-chain ceramide (CER) species or altered molar ratios among CER subclasses [12,13,14].

The progression of keratinocyte differentiation, which allows the stepwise acquisition of SC components, is tightly controlled by specific transcription factors (TFs), such as p63, Notch, AP-1, KLF4, DLX3 GATA3, EGR3, and OVOL1/2 [15,16,17,18,19,20,21,22]. The retinoic acid receptor-related orphan receptors (RORs: RORα, RORβ, and RORγ) are members of the nuclear receptor superfamily and serve as ligand-regulated transcription factors [23,24]. RORα functions are implicated in development, metabolism, immunity, and circadian rhythm [25,26]. Previous studies also showed the predominant expression of RORα in the skin epidermis in humans and mice [27,28]. In cultured human and mouse keratinocytes, RORα is a positive regulator of early and late differentiation [29,30]. Recent reports revealed that the epidermal RORα levels are significantly downregulated in clinical samples of several inflammatory skin diseases, including allergic contact dermatitis, lichen simplex chronicus, and sarcoidosis [31]. To address the significance of epidermal RORα, we recently generated a mouse strain with an epidermis-specific *Rora* deletion (*Rora^EKO^*), and we found that MC903-induced AD-like skin inflammation was greatly enhanced with an epidermal RORα deficiency [32]. In the current study, we wish to report the use of *Rora^EKO^* mice for characterizing the in vivo functions of RORα in regulating keratinocyte differentiation and epidermal barrier function.

## 2. Results

### 2.1. Rora^EKO^ Mice Display Elevated Transepidermal Water Loss and Aberrant Skin Phenotypes

**Figure 1:**
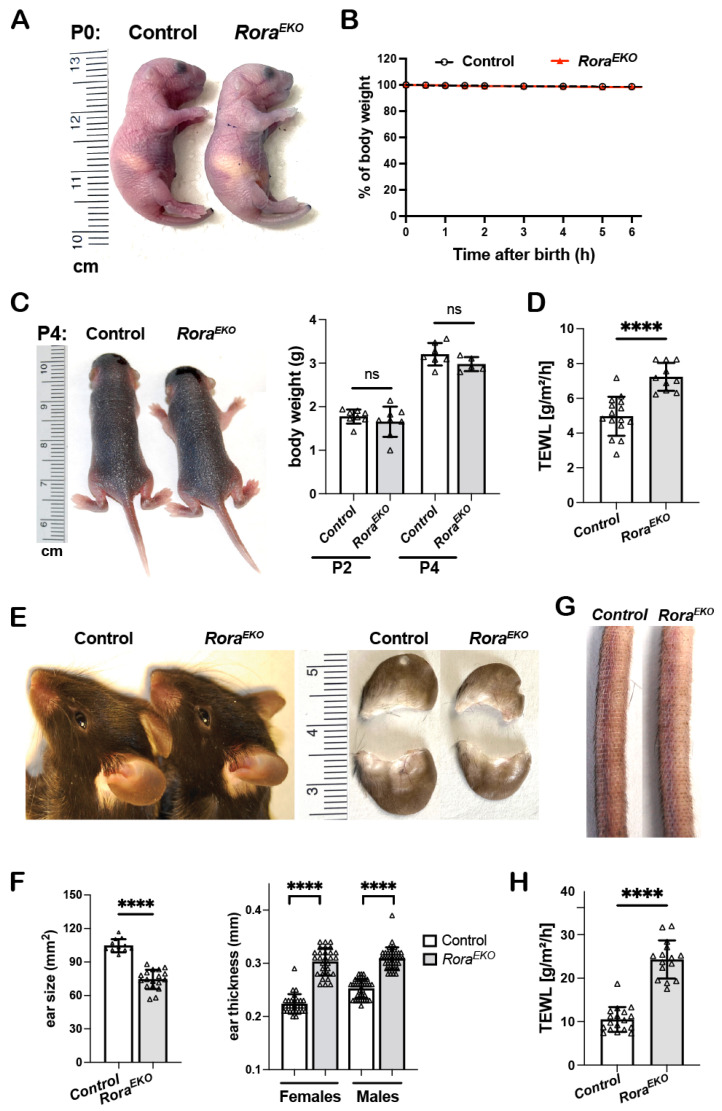
Disturbed barrier function and aberrant skin phenotypes in *Rora^EKO^* mice. (**A**) Gross appearance of P0 mice following a toluidine blue dye penetration assay. (**B**) The body weight of P0 mice was measured at room temperature over a 6 h period and presented as the percentages of initial weight, *n* = 7/genotype. (**C**) Gross appearance (left) and body weight (right) of P2 and P4 mice, *n* = 5–8/genotype. (**D**) TEWL rates across the back skin of P4 mice (*n* = 10–15/genotype). (**E**) Ear photos of 10-week-old mice. (**F**) Quantitation of ear size (*n* = 12–18/genotype) and ear thickness (*n* = 36–44/group). (**G**) Representative tail photo of 10-week-old mice. (**H**) TEWL across the shaved back skin of 8–10-week mice (*n* = 15–18/genotype)**.** All quantitative data are presented as mean ± SD; ns, not significant, or **** *p* < 0.0001 was determined by unpaired *t*-test. Each triangle represents one sample.

Similar to the control littermates, postnatal P0 *Rora^EKO^* mice with an epidermis-specific *Rora* deletion were resistant to toluidine blue penetration (Figure 1A) and did not show weight loss (Figure 1B). Between P2 and P4, the two strains remained indistinguishable regarding gross appearance and body weight (Figure 1C). However, the dorsal skin of neonatal *Rora^EKO^* mice displayed a significantly higher rate of transepidermal water loss (TEWL) than that of control mice (Figure 1D), indicating a compromised barrier function. Adult *Rora^EKO^* mice did not show overt skin lesions or spontaneous dermatitis. However, a closer examination could distinguish adult *Rora^EKO^* mice from their sex-matched littermates based on (a) significantly smaller but thicker ears (Figure 1E,F), (b) rough and dry tail skin (Figure 1G), and (c) an elevated TEWL rate across the shaved back skin (Figure 1H). Interestingly, these subclinical skin phenotypes were also described in mutant mice with a filaggrin deficiency or *Flg/Hrnr* double deletions, implying that an epidermal RORα loss may reduce CE structural proteins [33,34,35].

### 2.2. Epidermal Rora Gene Deletion Decreases the Expression of Late Differentiation Markers

**Figure 2:**
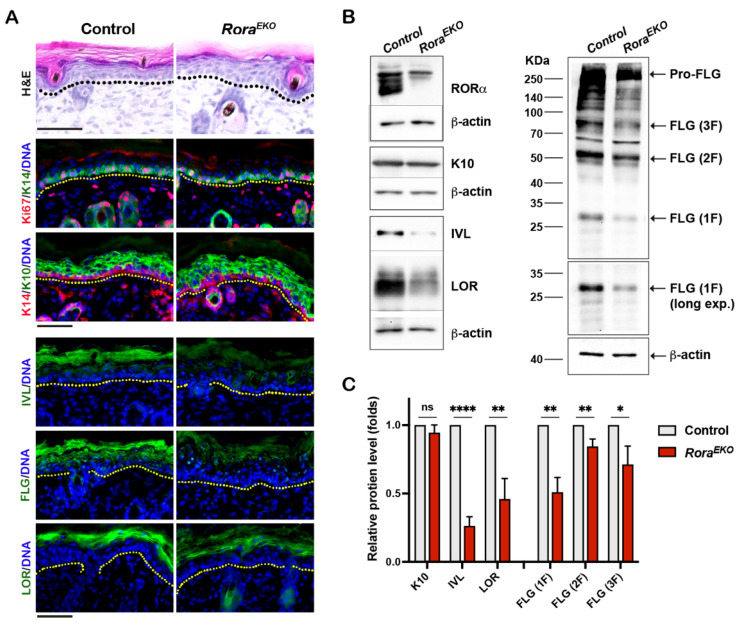
Epidermal *Rora* gene deletion downregulates markers of late keratinocyte differentiation. (**A**) Representative images of Hematoxylin and Eosin (H&E) staining (top) and immunostaining of indicated proteins on the frozen skin sections of P4 mice. Immunofluorescence images include keratin 6 (K6) + CD11c; keratin 10 (K10) + CD4; and Loricrin + F4/80. DNA was counterstained with Hoechst (blue). Dotted lines mark the epidermis/dermis junctions. Scale bars = 50 μm. (**B**) Representative Western blot gel images of indicated differentiation markers. (**C**) The levels of indicated proteins were quantified by densitometry scanning using ImageJ 1.53k and normalized to b-tubulin. Values are presented as mean folds of control ± SD, n = 3; ns, not significant, * *p* < 0.05, ** *p* < 0.01, or **** *p* < 0.0001 was determined by unpaired *t*-test.

Previous studies showed that RORα is predominantly expressed in mouse skin’s epidermis, hair follicle, and sebaceous glands [28]. An RORα signal was also detected in the dermis of normal human skin [29,31]. Compared to the control littermates, P4 Rora^EKO^ mice displayed normal skin thickness in each compartment, i.e., the epidermis, dermis, and subcutaneous adipose tissue (Supplementary Figure S1). However, the upper epidermis of Rora^EKO^ skin exhibited less compact granular layers and a less defined basket weave-like structure in the SC, in contrast with the well-organized structures in control mice (Figure 2A, H&E). Consistently, the intensities of involucrin (IVL), loricrin (LOR), and filaggrin (FLG) proteins were markedly decreased in the upper epidermis of Rora^EKO^ skin compared to the control skin, despite the normal levels of K14 and Ki67 in the basal layer and K10 in suprabasal layers (Figure 2A). Western blot analysis validated the significant reduction in IVL, LOR, the active FLG monomer, as well as pro-FLG and processed FLG dimers (2F) and trimers (3F) in the Rora^EKO^ epidermis vs. the control (Figure 2B,C; Supplementary Figure S2A–D). These results underline RORα’s in vivo importance for late keratinocyte differentiation.

### 2.3. Epidermal RORα Deficiency Alters Gene Expression Profiles

**Figure 3:**
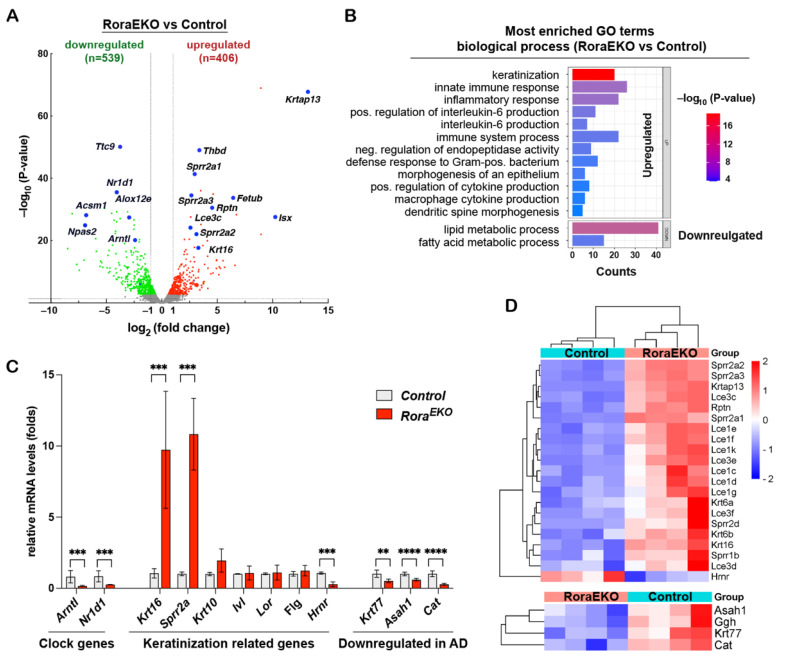
*Rora^EKO^* epidermis displays disturbed gene expression profiles. (**A**) Volcano plot of RNA-seq data indicating differentially expressed genes (DEGs) in *Rora^EKO^* versus control epidermis at P4 (n = 4/genotype). Red and green dots denote respective upregulated and downregulated expressions at fold-change ≥ 1.5 and adjusted *p* < 0.05. (**B**) Most enriched Gene Ontology (GO) terms among DEGs (*Rora^EKO^*/control; fold-change ≥ 1.5 and adjusted *p* < 0.05) from RNA-seq data, as revealed by DAVID 6.8 software (Benjamini *p* values < 0.05). (**C**) qRT-PCR validation of indicated DEGs. The mRNA level of each gene was normalized to *18S* and presented as mean-fold of control ± SD, n = 4–6/genotype; ** *p* < 0.01, *** *p* < 0.001, or **** *p* < 0.0001 was determined by unpaired *t*-test. (**D**) Heatmaps with hierarchical clustering showing differential expression of “keratinization” (top) and AD related genes (bottom) from RNA-seq data.

To better understand the downstream gene network of epidermal RORα, we performed an RNA sequencing (RNA-seq) transcriptomic analysis using the dorsal skin epidermis of P4 *Rora^EKO^* and control mice (n = 4/genotype). Differentially expressed genes (DEGs) were analyzed by the edgeR package [36]. Using fold changes > 1.5 and an adjusted *p* value < 0.05 as cutoffs, we identified 406 significantly upregulated genes and 539 downregulated genes in the *Rora^EKO^* epidermis vs. the control (Figure 3A; Supplementary Table S1). The Gene Ontology (GO) enrichment analysis of these genes using the DAVID 6.8 online tools reveals that *Rora^EKO^* downregulated genes were enriched in the GO terms of lipid and fatty acid metabolic processes (Figure 3B; Supplementary Tables S2 and S3). On the other hand, *Rora^EKO^* upregulated genes are strongly associated with the biological processes of keratinization, innate inflammatory response, positive regulation of interleukin-6 production, and negative regulation of endopeptidase activity (Figure 3B; Supplementary Tables S2 and S3).

Notably, three genes related to circadian rhythm were among the top 25 downregulated genes in the *Rora^EKO^* epidermis, including *Arntl* (Bmal1, Basic Helix-Loop-Helix ARNT Like 1) and *Npas2* (Neuronal PAS Domain Protein 2), which are known direct RORα targets, and *Nr1d1* (REV-ERBα), a competitive repressor of RORα activity (Figure 3A; Supplementary Table S1) [37,38,39]. An RT-PCR analysis validated the significant reduction in Arntl and *Nr1d1* mRNA levels in *Rora^EKO^* epidermis vs. control (Figure 3C), indicating that clock genes are common RORα targets among different tissues. The *Rora^EKO^* upregulated “keratinization” cluster consisted of several stress-response genes (*Krt16*, *Krt6a*, and *Krt6b*) and numerous genes in the epidermal differentiation complex (EDC), which is a dense cluster of over 60 genes located in human chromosome 1q21 and mouse chromosome 3q [40,41,42]. *Rora^EKO^* upregulated EDC genes included *Rnpn* (Repetin) and multiple members of the late cornified envelope (*Lce*) and the small proline-rich (*Sprr*) gene families (Figure 3C,D). In contrast, the transcription level of *Hrnr* (hornerin), an *FLG*-like EDC gene, was markedly reduced in the *Rora^EKO^* epidermis compared with control (Figure 3C,D). Despite the considerable reduction in involucrin, loricrin, and filaggrin in the *Rora^EKO^* epidermis (Figure 2A–C), the RNA-seq and RT-PCR analysis failed to detect altered mRNA levels of these EDC genes (*Ivl*, *Lor*, and *Flg*) (Figure 3C). These results indicate that epidermal RORα is essential for coordinated gene expression on the EDC locus, the center of keratinocyte terminal differentiation [40].

Interestingly, the RNA-seq analysis also revealed a substantial reduction in Krt77, *Asah1*, *Cat, and Ggh* genes in the *Rora^EKO^* epidermis vs. control (Figure 3D, lower panel), which was further validated using an RT-PCR analysis (Figure 3C). The products of these genes are among the nine signature proteins significantly decreased in the skin tape strip (STS) from patients carrying atopic dermatitis and food allergies compared to the healthy subjects [43]. Therefore, an epidermal *Rora* deficiency leads to a gene signature related to allergic diseases.

### 2.4. Epidermal Rora Gene Deletion Alters Ceramide Composition in the Epidermis

**Figure 4:**
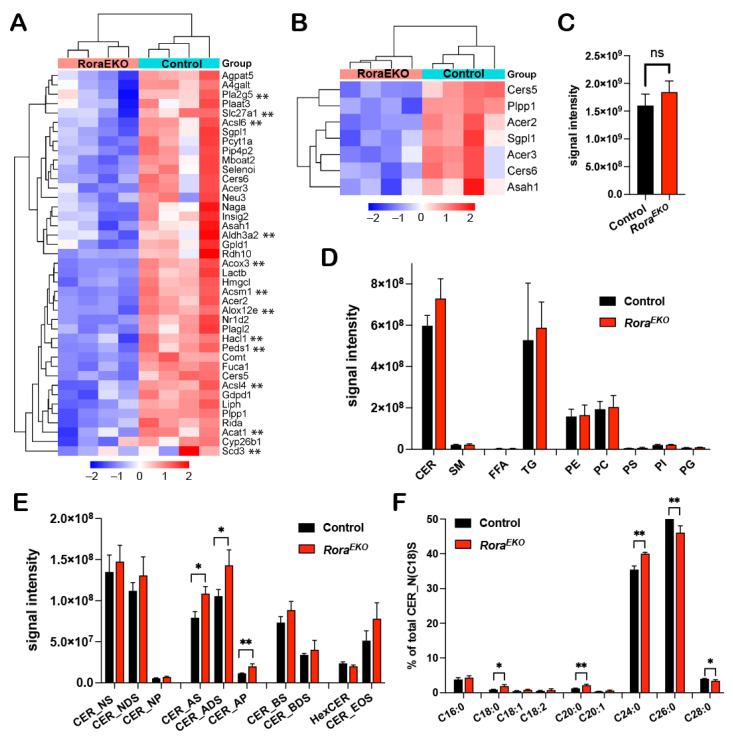
Disturbed lipid composition in the *Rora^EKO^* epidermis. (**A**,**B**) Heatmaps with hierarchical clustering showing differential expression of “lipid metabolic process” (**A**) and “ceramide metabolism” related genes (**B**) from RNA-seq data. (**C**–**F**) LC-MS/MS analysis of lipids in P4 mouse epidermis. Data show signal intensity of total lipids (**C**), major lipid classes (**D**), and CER subclasses (**E**). (**F**) Percentage of CERs (with variable FA carbons) within the CER_NS subclass containing C18 sphingosine. Data were presented as mean values ± SD, n = 3/genotype; ns, not significant, * *p* < 0.05, or ** *p* < 0.01 was determined by unpaired *t*-test.

As revealed by the RNA-seq analysis, 41 out of the 409 significantly downregulated genes (*Rora^EKO^* vs. control) are related to the “lipid metabolic process” (Figure 3B and Figure 4A; Supplementary Table S3), with subclusters specifically related to fatty acid metabolism (Figure 4A, genes with **) and ceramide metabolism (Figure 4B). To evaluate the direct impact of RORα loss on epidermal lipid metabolism, we performed an LC-MS/MS lipidomic analysis. In this study, 678 and 465 lipid species were detected in the epidermis tissue of P4 mice by the negative and positive ion modes, respectively. There was no significant difference between *Rora^EKO^* and the control epidermis in terms of the total lipid amount (Figure 4C) or the abundance of each major lipid class, including ceramide (CER), sphingomyelin (SM), free fatty acids (FFAs), triglyceride (TG), and various phospholipids, i.e., phosphatidyl-ethanolamine, -choline; -serine, -inositol; -glycerol (PE, PC, PS, PI, PG) (Figure 4D).

Ceramides are the major lipids in the SC of normal skin, and each molecule comprises a sphingoid base and a fatty acid via an amide bond [44]. The combination of different structures in the two moieties leads to diverse CER subclasses with a wide range of carbon chain lengths. We found that the levels of the CER subclasses containing alpha-hydroxy FAs (A) were significantly higher in the *Rora^EKO^* epidermis compared to the control (Figure 4E). These included CER_AS, CER_ADS, and CER_AP, for which the alpha-hydroxylated FAs were coupled with sphingosine (S), dihydrosphingosine (DS), or phytosphingosine (P), respectively (Figure 4E). In contrast, no significant differences were observed with the non-hydroxy CERs [NS, NDS, NP], beta-hydroxy CERs (BS, BDS), hexosylceramides (HexCERs), and esterified omega-hydroxy FA (EOS) (Figure 4E). Beta-hydroxy CERs were only detected in the SC of mouse skin [45]. Importantly, within the CER_NS subclass containing a C18 sphingoid base (CER_N(C18)S), the percentages of CERs containing C18–C24 FAs were significantly increased in the *Rora^EKO^* epidermis vs. the controls. In contrast, C26 and C28 CERs’ percentages decreased (Figure 4F). In contrast, no significant change was observed with FFAs and triglyceride (TG) subspecies between the two genotypes (Supplementary Figure S3). These results underscore the importance of epidermal RORα for a balanced CER composition in mouse skin.

### 2.5. Oxazolone-Induced Percutaneous Inflammation Is Enhanced in Rora^EKO^ Mice

Adult *Rora^EKO^* mice displayed a higher rate of TEWL without an apparent sign of dermatitis compared with the control littermates (Figure 1E,H). To determine whether *Rora^EKO^* mice are more susceptible to percutaneous immune responses to external stimuli, we employed a contact hypersensitivity (CHS) mouse model widely used for studying molecular mechanisms of allergic contact dermatitis (ACD) [46]. ACD is a T-cell-mediated hypersensitivity reaction to a hapten and occurs in two sequential phases: percutaneous sensitization and elicitation [47]. In this model, mice were first sensitized on the shaved abdomen skin with a classical dose (3%) of oxazolone (Oxa). Five days later, the right ear was topically challenged with a threshold dose (0.3% *v*/*v*) of Oxa to initiate the elicitation phase, and the left ear was treated with vehicle (EtOH) as a control [33].

**Figure 5:**
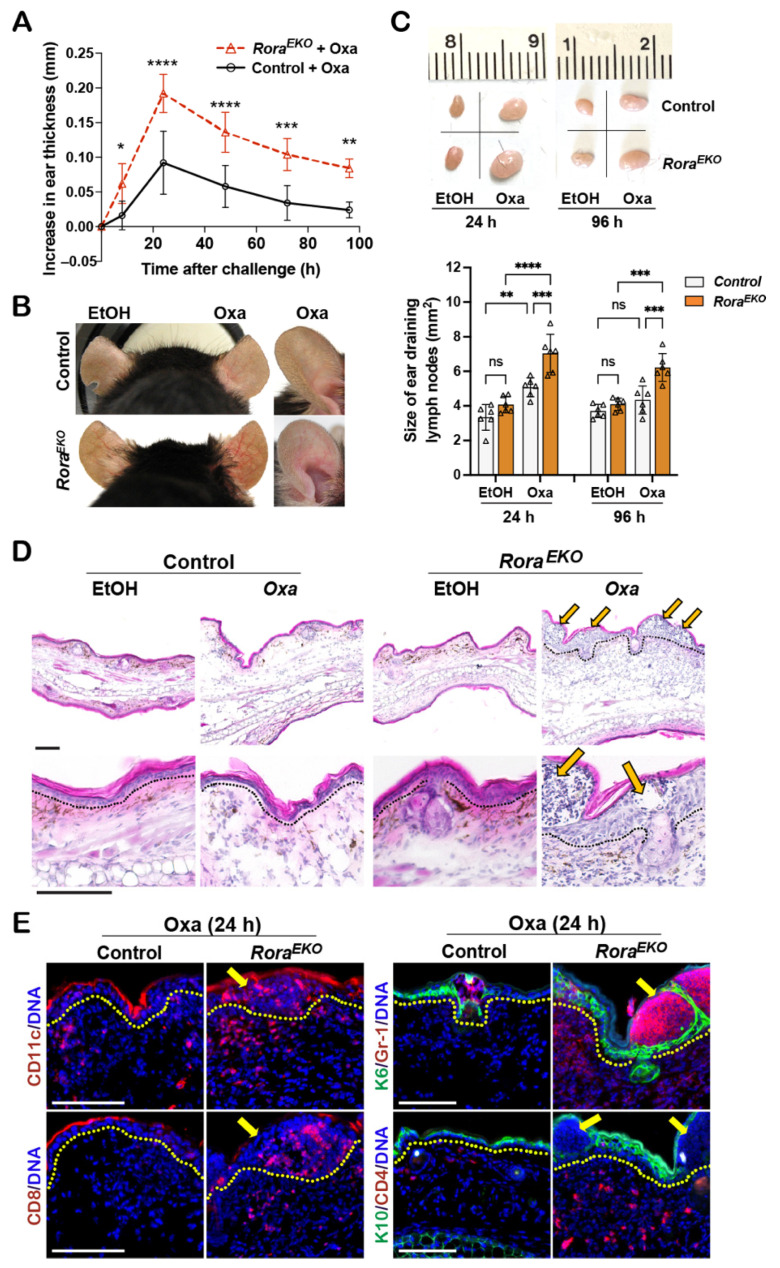
*Rora^EKO^* mice mount robust inflammation in oxazolone-induced CHS model. (**A**) Mouse-ear thickness was measured daily after Oxa-elicitation and plotted as the increase in ear thickness; n = 5/genotype. (**B**) Representative ear photos at 24 h after elicitation. (**C**) Top: Representative photos of ear-draining lymph nodes (dLNs). Bottom: The sizes of ear dLNs measured by ImageJ 1.53k and presented as average size ± SD n = 5/group; each triangle represents one sample; ns, not significant, * *p* < 0.05, ** *p* < 0.01, *** *p* < 0.001, or **** *p* < 0.0001 was determined by two-way ANNOVA for (**A**) or one-way ANNOVA for (**C**). (**D**) H&E images of ear sections collected at 24 h elicitation. Arrows indicate spongiotic sites. (**E**) Representative immunofluorescence images of indicated proteins in ears with 24 h Oxa-elicitation, including CD11c (red), CD8 (red), keratin 6 (K6, green) + Gr-1 (red), and keratin 10 (K10, green) + CD4 (red). DNA was counterstained with Hoechst (blue). Dotted lines in (**D**,**E**) mark the epidermis/dermis junctions; scale bar = 100 μm.

While EtOH did not affect ear thickness, the Oxa challenge induced ear swelling, peaking at 24 h in both types of mice (Figure 5A). As compared to the sex-matched control littermates, *Rora^EKO^* mice exhibited robust ear thickening (Figure 5A), apparent vasodilation in the ear skin (Figure 5B), and enhanced enlargement of ear-draining lymph nodes (Figure 5C), which are characteristics of exacerbated inflammation. Histologically, the Oxa-challenged ears of control mice showed a mild dermal expansion and an overall normal epidermis compared to EtOH-treated ears (Figure 5D). In contrast, Oxa-treated *Rora^EKO^* ear skin exhibited robust expansion and heavy immune infiltration at the dermis and epidermis (Figure 5D). Strikingly, the expanded epidermis of *Rora^EKO^* mice was occupied with fluid-containing large cysts, the clinical manifestations of spongiosis (intercellular edema) (Figure 5D, arrows). Immunostaining revealed an intensive epidermal induction of K6, along with the massive infiltration of CD11c+ dendritic cells, Gr1+ neutrophils, CD8+ T cells, and CD4+ cells in Oxa-challenged *Rora^EKO^* ear skin, a direct contrast with the minimal infiltration of respective cell types in control mice (Figure 5E; arrows indicate spongiotic sites). These results suggest that epidermal RORα loss can exacerbate cutaneous allergic responses by enhancing the local recruitment of both innate and adaptive immune cells.

## 3. Discussion

The present study has identified epidermal RORα as a central regulator required for normal barrier function in vivo. The epidermal RORα deficiency increased the TEWL rate at a steady state and exacerbated skin inflammation in the ACD mouse model. The compromised skin barrier function in *Rora^EKO^* mice could be attributed to the deficient expression of key CE proteins, aberrant ceramide profiles, and altered gene expression profiles. These findings substantiate the multiple functions of epidermal RORα in late differentiation and barrier maintenance.

### 3.1. In Vivo Role of RORα on Late Keratinocyte Differentiation

Previously, we found that siRNA-mediated *RORA* knockdown in human keratinocytes significantly reduced differentiation markers at all stages [29]. Here, the prominent defects in the *Rora^EKO^* epidermis appeared to concentrate at the upper epidermal compartment, based on the significant reduction in key CE proteins such as involucrin, loricrin, and filaggrin. Furthermore, the skin phenotypes of adult *Rora^EKO^* mice are reminiscent of mice with an *Flg* deficiency or *Flg*/*Hrnr* double deletions [33,34,35] (Figure 2B,C), providing additional evidence for the in vivo importance of RORα in CE protein expression and late differentiation.

The molecular mechanisms regarding how RORα drives the expression of key CE genes remain unknown to date. A recent study using mouse suprabasal keratinocytes reported that H3K27Ac bound DNA peaks, which correspond to active enhancer regions, are enriched for binding motifs of ROR, GRHL, KLF, and DLX [17]. In the same study, ROR binding motifs are also present in the DNA peaks bound by Dlx3, a well-known pro-differentiation transcription factor (TF) that directly binds to the regulatory regions of many EDC genes, such as *Hrnr*. These findings indicate that RORα may directly or indirectly drive the expression of CE proteins encoded by the EDC locus via its interaction with other TFs, such as Dlx3.

### 3.2. Role of Epidermal RORα in Regulating Ceramide Metabolism

Besides losing key CE proteins, the *Rora^EKO^* epidermis also displayed aberrant ceramide (CER) profiles featuring an increased abundance of alpha-hydroxy subclass (Figure 4E). Higher levels of CER_AS, CER_ADS, and CER_AP were found in keratinocytes from patients with psoriasis compared to the healthy controls [48]. However, it is unclear how elevated hydroxylated CERs could contribute to barrier dysfunction. Nevertheless, Emmert et al. reported that CER_AS and CER_ADS levels have a positive correlation with *staphylococcus aureus* colonization in AD skin lesions [49], indicating that epidermal RORα may have additional functions in maintaining balanced skin microbiome composition.

It is noteworthy that *Rora^EKO^* downregulated “ceramide metabolism” genes are mainly involved in the recycle and salvage pathways in sphingolipid metabolism (Figure 4B) [50]. Among these genes, *Asah1*-encoded acid ceramidase (AC) and *Acer2* and *Acer3*-encoded alkaline ceramidases-2 and -3 are responsible for breaking CERs into sphingosine and fatty acid, whereas *Cers5* and *Cers6* encoded CER synthases catalyze the reverse reaction [51,52,53]. In human keratinocytes, a knockdown of *ASAH1* attenuates Ca^2+^-induced keratin 1 and involucrin expression. The blocked differentiation in *Asah1* deficient cells could be due to the reduction in sphingosine and sphingosine-1-phosphate, which are the signaling molecules promoting growth arrest and differentiation [54,55]. Future studies will investigate how a reduced expression of *Asah1* and other genes in this pathway may contribute to the defective differentiation program or aberrant CER profiles, especially the accumulation of hydroxy CERs in RORα deficient epidermis.

In addition to elevated alpha-hydroxy CERs, the *Rora^EKO^* epidermis showed increased percentages of shorter-chain and decreased long-chain species within the CER_NS subclass (Figure 4F). Interestingly, similar alternations were also observed in the stratum corneum of AD lesions in both human and IL-13 transgenic mice compared to the healthy controls. At the molecular level, such changes are associated with the decreased expression levels of the very long chain FA elongases, ELOVL3 and ELOVL6 [56]. In the current study, an RNA-seq analysis did not reveal a significant change in *Elovl* genes in the *Rora^EKO^* epidermis vs. the control. Moreover, the shift of the acyl chain lengths was not observed with other lipid classes such as free FAs (FFAs) or triglycerides (TGs) (Supplementary Figure S3).

The lack of profound change in FFA and TG species in the *Rora^EKO^* epidermis is likely due to the functional redundancy between RORα and RORγ isoforms, which has been demonstrated in other tissues. In mouse liver, the significant changes in most lipid metabolism-related genes, including *Elovl3*, were only detected when both isoforms were depleted [57,58]. It is plausible that RORα and RORγ may also have functional redundancy in regulating certain gene subsets in the epidermis, leading to transient and mild phenotypes with a *Rora* single deletion.

### 3.3. Role of Epidermal RORα in Preventing Skin Inflammation

Like filaggrin-deficient mice [33,59,60], *Rora^EKO^* mice exhibited enhanced cutaneous responses at a threshold Oxa-elicitation concentration in the CHS mouse model (Figure 5). This finding confirmed the barrier impairment of *Rora^EKO^* skin at the functional level. Accordingly, we expect that the major epidermal RORα reduction in skin lesions of diverse inflammatory skin diseases can amplify contact allergy and accelerate disease progression [31]. Epidermal RORα loss may also represent a strong risk factor for chronic allergic inflammatory diseases such as AD. First, such loss downregulated key CE proteins like filaggrin, loricrin, and involucrin, typically decreased in AD lesions due to gene mutations or suppression by Th2 cytokines [10,61,62]. Second, four out of nine signature proteins downregulated in patients with AD and food allergies (i.e., *Krt77*, *Asah1*, *Ggh*, *Cat*) were significantly decreased in the *Rora^EKO^* epidermis vs. the control (Figure 3C,D), indicating RORα could be critical for maintaining the expression levels of these important genes [43]. At the same time, the RORα deficiency upregulated multiple gene clusters related to inflammatory responses (Figure 3B; Supplementary Tables S2 and S3). Therefore, epidermal RORα may have dual actions in preventing skin inflammation by maintaining barrier integrity and suppressing the innate immune response of keratinocytes.

Lastly, an examination of gene expression profiles reveals overlapping and unique targets between RORα and other transcription factors with similar functions. For instance, the upregulated “keratinization” genes (e.g., *Krt6*, *Krt16*, *Sprr2d*, *Sprr2h*) shown in *Rora^EKO^* epidermis (Figure 3C,D) are also increased in mutant mice lacking *Gata3*, *Klf4*, or *ZFP750*, the mouse homolog of human *ZNF750* [20,21,63]. The upregulation of these genes likely reflects a compensatory response to barrier dysfunction, the common feature among these mutants [20,21,63]. Meanwhile, although both *Rora^EKO^* and *ZEP750^−/−^* epidermis exhibited a downregulation of many genes related to lipid metabolism, only a few overlapping genes were found between the two groups (Figure 4A) [63]. Similarly, while an epidermal RORα deficiency downregulated *Asah1*, *Acer2,* and *Acer3* genes, loss of COUP-TF interacting protein 2 (Ctip2) caused a great reduction in *Asah2* and *Acer1* [64]. These findings imply that RORα and other TFs may coordinate to regulate different subsets of genes in promoting terminal differentiation.

In conclusion, we have demonstrated that epidermal RORα maintains normal barrier integrity and prevents inflammation by regulating CE structural proteins and lipid composition. A major limitation of the current study is that the molecular mechanisms underlying epidermal RORα’s functions remain unknown. Additionally, although RNA-seq analysis revealed numerous genes and pathways altered by epidermal RORα loss, the functional relevance of these changes is still missing. Future studies will employ a Chromatin Immunoprecipitation (ChIP) analysis to identify the direct and indirect targets of RORα in keratinocytes and identify its network in orchestrating the terminal differentiation process. In vitro and in vivo studies are needed to uncover the specific roles of RORα target genes in skin barrier function and skin inflammation, focusing on the genes related to pathological conditions. Furthermore, given the marked reduction in numerous clock genes in the *Rora^EKO^* epidermis (Figure 3A,C), it is imperative to understand the role of epidermal RORα in regulating circadian rhythms of barrier function and keratinocyte innate immunity. Importantly, these findings ascertain the beneficial potentials of RORα-selective agonists in restoring epidermal structural components and barrier functions for treating AD and other inflammatory skin diseases [65,66].

## 4. Materials and Methods

### 4.1. Mice

All animal studies were conducted under approved protocols from the Institutional Animal Care and Use Committee at the University of Wisconsin-Madison. As previously described [32], mice with an epidermis-specific Rora deletion (*Rora^EKO^*) were established by crossing the *Rora^flox/flox^* strain with the *K14-Cre* strain (B6N.Cg-Tg(KRT14-cre), (The Jackson Laboratory, Bar Harbor, ME, USA).

### 4.2. Dye Penetration Assay

As previously described [67], P0 mice were sacrificed and rinsed in PBS, followed by dehydration with 25%, 50%, 75%, and 100% of methanol/PBS. After rehydration with the same methanol solution series in the reverse order, mice were rinsed with PBS, immersed in 0.1% of toluidine blue/PBS for 10 min, and washed with PBS. Images were captured with a Nikon digital camera.

### 4.3. Body Weight Loss Assay

The body weight of P0 mice was monitored every 30 min or 1 h at room temperature over 6 h.

### 4.4. Measurement of Transepidermal Water Loss (TEWL)

The dorsal skin of 8–10-week mice was shaved two days before the measurements. TEWL rates were measured on the shaved dorsal skin of adult mice or unshaved skin of P4 pups with the Tewameter TM Hex probe (Courage and Khazaka Electronic GmbH, Cologne, Germany), according to the manufacturer’s instructions.

### 4.5. Measurement of Ear Size and Ear Thickness

The ear thickness of adult mice was measured with a digital caliper (Mitutoyo Corp., Tokyo, Japan). After excision, the ear size was measured using ImageJ software (National Institute of Health).

### 4.6. Oxazolone-Induced Contact Hypersensitivity (CHS): Acute Contact Dermatitis (ACD) Model

On day 0, mice of 8–10 weeks were shaved on the abdomen. The mice were then sensitized by an epicutaneous application with 100 µL of 3% oxazolone or Oxa (4-ethoxymethylene-2-phenyl-2-oxazolin-5-one from Sigma-Aldrich, Saint Louis, MO, USA) in ethanol onto 2 cm^2^ of the shaved skin. On day 5, the right ear skin was challenged by a topical application of a non-irritant dose of oxazolone (0.3%, 10 µL on each side), whereas the left ear received the same volume of vehicle. Ear thickness was measured with a digital caliper (Mitutoyo Corp., Tokyo, Japan) before (0 h) and at different time points after the challenge. Mice were euthanized at 24 h or 96 h post elicitation to collect ear tissues for a histological analysis. Ear-draining lymph nodes (dLNs) were dissected, and the size of dLNs was quantified by the ImageJ software.

### 4.7. Histological and Immunofluorescence Analysis

Frozen sections (10 μm) of back skin or ear samples were fixed with 10% formalin for Hematoxylin & Eosin Y (H&E) staining (Electron Microscopy Sciences, Hatfield, PA, USA) using standard protocols. For immunostaining, frozen sections (10 μm) were fixed with 4% paraformaldehyde/PBS and permeabilized with 0.1% NP-40/PBS for 15 min before overnight incubation with primary antibodies at 4 °C. Slides were then incubated with Alexa 488- or Alexa 594-conjugated secondary antibodies (Invitrogen-Thermo Fisher Scientific, Waltham, MA, USA). Hoechst 33342 (Pierce Biotechnology-Thermo Fisher Scientific) was used for DNA detection. H&E and fluorescence images were acquired with the Lionheart FX microscope (Biotek, Winooski, VT, USA). A list of primary antibodies for immunostaining is provided in Supplementary Materials and Methods.

### 4.8. Isolation of Epidermis from Mouse Back Skin

The back skin of P4 pups was subjected to a 30-sec heat treatment in PBS preheated to 60 °C, followed by a 1 min cooling in ice-cold PBS. The epidermis was peeled from the dermis. After snap-freeze in liquid nitrogen, the frozen epidermis was stored at –80 °C for RNA isolation, protein extraction, or lipid extraction.

### 4.9. Western Blot Analysis

The separated epidermis was dissociated with a homogenizer (Fisherbrand Bead Mill 4, Fisher Scientific, Hanover Park, IL, USA) in the protein lysis buffer containing 4% SDS, 10% 2-mercaptoethanol, 20% glycerol, and 0.125 M Tris-HCl. Protein concentration was determined by a Pierce BCA Protein Assay Kit (Thermo Fisher Scientific). Proteins were separated by SDS-polyacrylamide gel electrophoresis and transferred onto the 0.45-μm polyvinylidene difluoride membrane (Thermo Fisher Scientific). After blocking with 5% non-fat milk, membranes were incubated with the specific antibodies overnight at 4 °C, followed by incubation with the horseradish peroxidase (HRP)-conjugated secondary antibodies (Jackson ImmunoResearch Laboratories). Membranes were incubated with the ECL Western blotting substrate (Pierce Biotechnology-Thermo Fisher Scientific). Chemiluminescent images were acquired with the iBright CL1000 (Invitrogen-Thermo Fisher Scientific, Pittsburgh, PA). The ImageJ software was used to quantify the relative protein expression levels. A list of primary antibodies for Western blotting is provided in Supplementary Materials and Methods.

### 4.10. RNA Transcriptomics and Gene Set Enrichment Analysis

The epidermis tissue from P4 mice was minced and homogenized in RLT buffer, using a Bead Mill 4 (Fisher Scientific, Hanover Park, IL, USA). Total RNA was isolated with the RNeasy mini kit (Qiagen, Germantown, MD, USA). We used the University of Wisconsin-Madison Biotechnology Center’s Gene Expression Center Core Facility (research resource identifier [RRID]: SCR_017759) for RNA quality control, preparation of TruSeq Stranded mRNA library (for polyA enrichment), and RNA sequencing using the Illumina NovaSeq 6000 platform. Raw sequencing data were converted with Illumina bcl2fastq v2.20.0.422; read trimming was conducted with Skewer v0.2.2b [68]. The trimmed paired-end reads were mapped to the Mus musculus reference genome (GRCm39) using STAR v2.5.3a software [69]. Mapped paired-end read estimates were counted in each sample using RSEM v1.2.31 [70]. Differential expression estimations (DEGs) were determined with a regularized logarithm transform using the edgeR package v3.28.0 [36]. Hierarchical clustering and heatmaps were generated with the SRplot free online tool [71]. DEGs with change >1.5-fold and a Benjamani–Hochberg adjusted *p* < 0.05 were used as cutoffs for the gene ontology analysis using the DAVID 6.8 online tools [72].

### 4.11. Quantitative Real-Time (RT)-PCR

Total RNA isolated from the epidermis, as described in Section 4.10., was reverse-transcribed with the High-Capacity cDNA RT Kit (Applied Biosystems-Thermo Fisher Scientific, Foster City, CA, USA). PCR reactions were performed with PowerSYBR Green PCR Master Mix using a QuantStudio3 Real-Time PCR system (Applied Biosystems-Thermo Fisher Scientific). The expression levels of target genes were calculated using the comparative method for relative quantification (2^−ΔΔ^ cycle threshold) and normalized to 18S rRNA. Each sample was tested in duplicates. Primer sequences for the RT-PCR analysis are listed in Supplementary Materials and Methods.

### 4.12. Lipid Analysis

The lipid extraction protocol is extensively based on Matyash’s method [73]. The weighted epidermis tissue was mixed with 250 µL of phosphate-buffered saline (PBS), 10 µL of internal standard mix, and 215 µL of methanol in bead-beating tubes and then subjected to 4 cycles of homogenization with TissueLyzer II (Qiagen). Samples were homogenized for 2 more cycles after mixing with 750 µL of MTBE (methyl tert-butyl ether). After centrifugation, the upper MTBE phase was lyophilized and reconstituted in isopropyl alcohol (IPA). For data acquisition, lipid samples in IPA were analyzed using UHPLC/MS and UHPLC/MS/MS in negative and positive ion modes. The assignment of lipid identities to mass and retention time signal pairs was conducted using Lipid Annotator (Agilent, Santa Clara, CA, USA) [74] and the LC/MS/MS data. Additional details for lipid analysis can be found in Supplementary Materials and Methods.

### 4.13. Statistical Analysis

All statistical evaluations used Prism 10.3.1 (GraphPad La Jolla, CA, USA). An unpaired Student’s *t*-test was used to compare the statistical difference between the two groups. A one-way ANOVA or two-way ANOVA analyzed the statistical differences between multiple groups, and *p* values < 0.05 were considered significant.

## Data Availability

Datasets related to this article are deposited in the Gene Expression Omnibus database under accession code (GSE275323). The data from the lipidomic analysis will be provided upon request.

## Supplementary Materials

The following supporting information can be downloaded at: https://www.mdpi.com/article/10.3390/ijms251910698/s1.
