# Supplementary material for: Epidermal RORα Maintains Barrier Integrity and Prevents Allergic Inflammation by Regulating Late Differentiation and Lipid Metabolism
Source: Int J Mol Sci. 2024 Oct 4;25(19):10698. doi: 10.3390/ijms251910698 (PMC11476758; doi:10.3390/ijms251910698)
Supplement: Supplementary file 1 [file ijms-25-10698-s001.zip › Supplementary information/Dai-Hua IJMS-3209820 Supplementary information.pdf]

SUPPLEMENTARY INFORMATION

Title: Epidermal RORα maintains barrier integrity and prevents allergic inflammation by regulating late differentiation and lipid metabolism

Xiangmei Hua,[1] Maria K. Ficaro,[1] Nicole L. Wallace,[1] and Jun Dai [1,2,3*]

[1] School of Pharmacy, The University of Wisconsin, Madison, WI 53705

[2] Carbone Cancer Center, The University of Wisconsin, Madison, WI 53705

[3] Skin Disease Research Center, The University of Wisconsin, Madison, WI 53705

* Corresponding author:
Jun Dai, PhD
Assistant Professor
The School of Pharmacy
University of Wisconsin, Madison
777 Highland Avenue
Madison, WI 53705
Tel: 608-292-3196
Email: jdai32@wisc.edu

Supplementary Figure S1

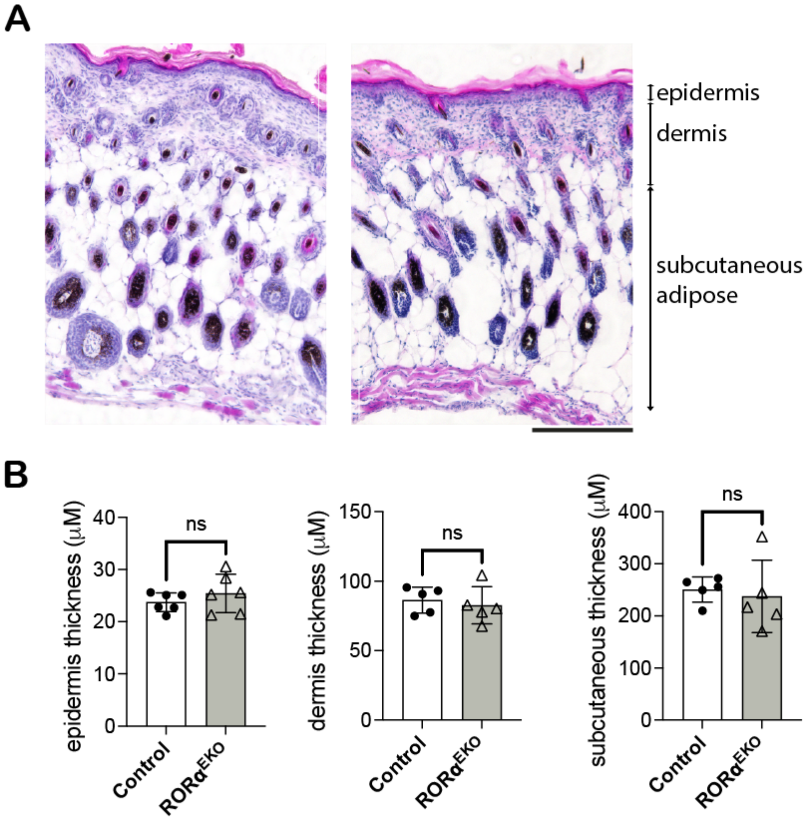

Supplementary Figure S1. ***Rora^{EKO}*** **skin displayed normal epidermal, dermal, and subcutaneous thickness.** (**A**) Hematoxylin and Eosin (H&E) stained frozen skin sections of P4 mice; bar = 200 μm. (**B**) The skin thickness was measured under 20x magnification (for epidermal thickness) or 4x magnification (for dermal and subcutaneous thickness) using ImageJ. The average thickness of each sample was quantified from at least 10 independent fields. Data show the mean average thickness ± SD, n=5 mice/group; ns, not significant, determined by an unpaired *t*-test.

Supplementary Figure S2

Supplementary Figure S2A. Original WB gel images for Figure 2B: RORα

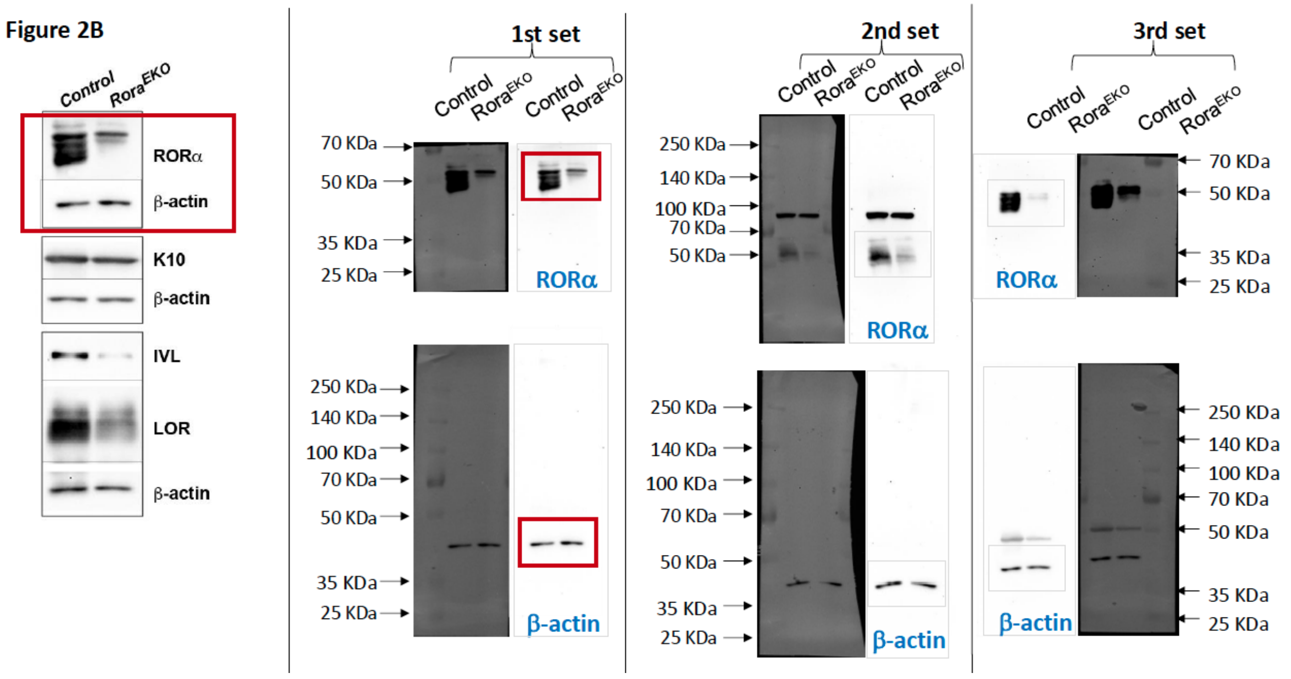

Supplementary Figure S2A. Original WB gel images for Figure 2B, 2C: K10

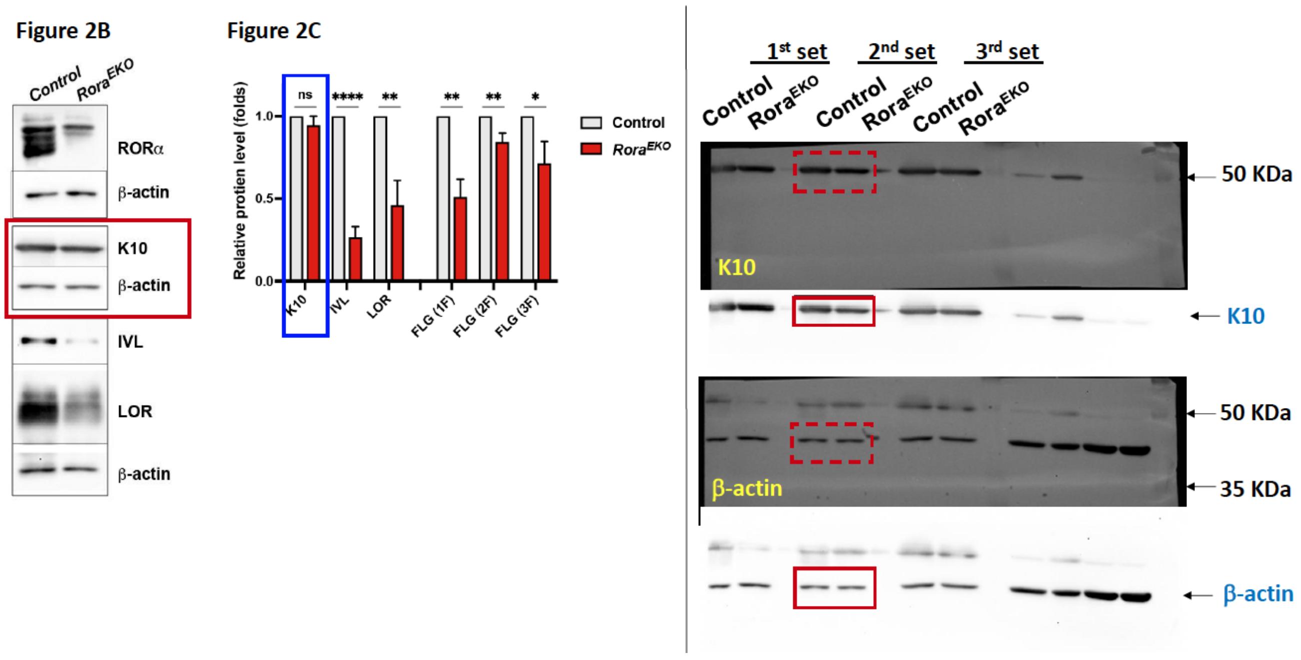

Supplementary Figure S2C. Original WB gel images for Figure 2B, 2C: IVL and LOR

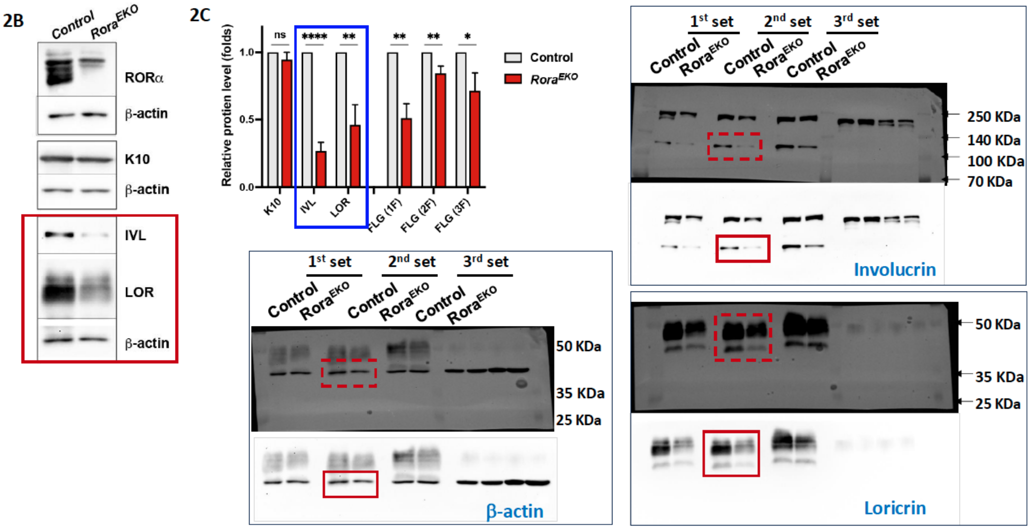

Supplementary Figure S2D. Original WB gel images for Figure 2B, 2C: FLG

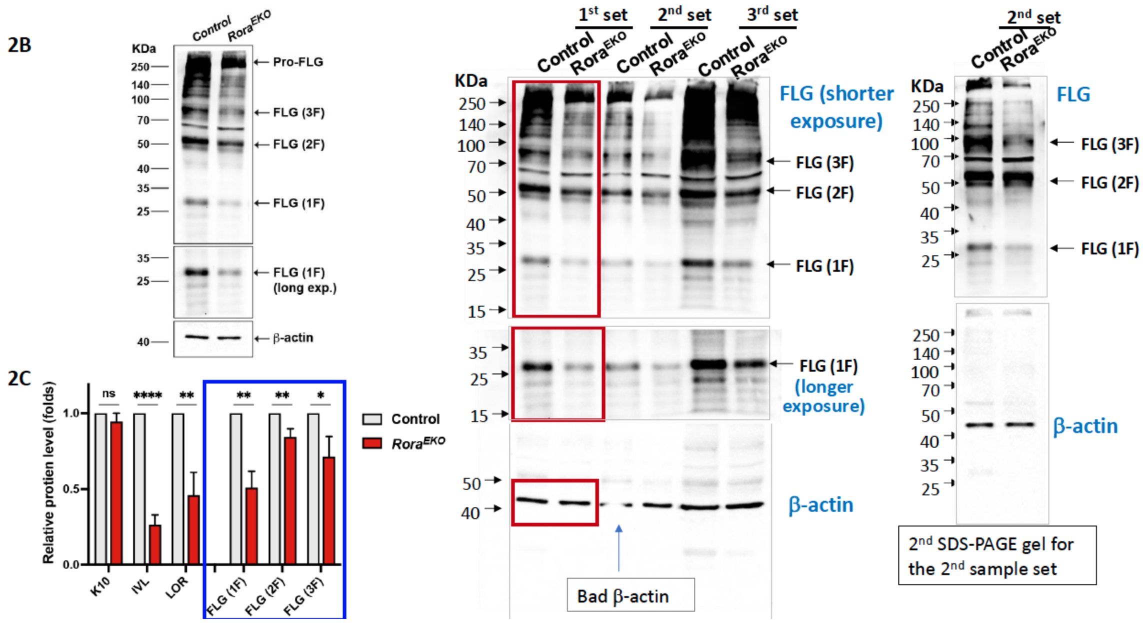

Supplementary Figure S2. Original protein gel images of western blot analysis for Figure 2B and 2C in the main text. Protein samples were collected from the epidermis tissue of P4 control or *Rora*^{EKO} mice. For each gel, the darker image shows the membrane overlay, and the lighter image shows the chemiluminescence signal. Red frames mark the representative images shown in the main text. Blue frames mark the relevant protein levels quantified from densitometry scanning of the three sets of samples.

Supplementary Figure S3

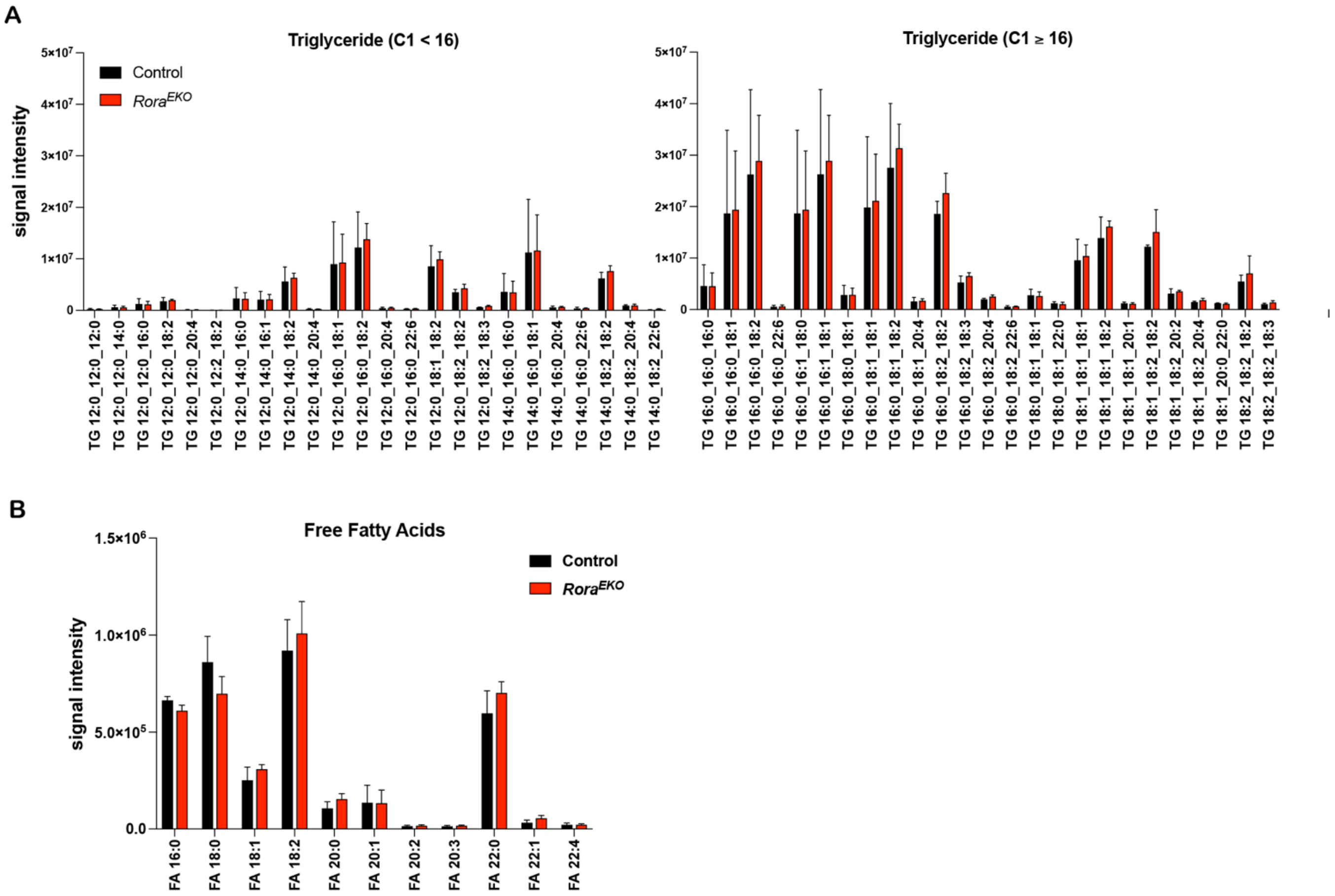

Supplementary Figure S3. ***Rora*^{EKO} skin displayed normal compositions of free fatty acids (FFAs) and triglycerides (TGs). (A-B)** LC-MS/MS analysis of lipids in P4 mouse epidermis. Data show the average signal intensity of individual FFAs (**A**) and TGs (**B**) and are presented as mean values ± SD, n=3/genotype.

SUPPLEMENTARY MATERIALS AND METHODS

Lipid analysis.

a. Sample extraction. The lipid extraction protocol is based extensively on Matyash's method [1]. Lipids are extracted in a solution comprising 250 µL of phosphate-buffered saline (PBS), 225 µL of methanol containing internal standards, and 750 µL of methyl tert-butyl ether (MTBE). The internal standard mix contains Avanti SPLASH LipidoMix (cat. #330707-1EA) at 10 µL per sample) supplemented with the additional standards from Cayman Chemical (Ann Arbor, MI), including C18 ceramide-d7 (d18:1-d7/18:0, cat. #22788), EOS-d9 (d18:1-d9/32:0/18:2, cat. #24423), linoleic acid-d11 (cat. #9002193), and heptadecanoyl-L-carnitine-d3 (cat #35459). Typically, tissues are weighed and placed in bead-beating tubes (Qiagen PowerBead tube containing 1.4mm ceramic beads, cat. # 13113-50), to which the PBS and methanol are added with internal standards.

In the current experiment, sections of epidermis tissue, held on dry ice, were transferred to bead-beating tubes and weighed. To each sample was added 250 µL of PBS, 10 µL of internal standard mix, and 215 µL of methanol. The methanol containing internal standards had sufficient volume for all the samples. Samples were then subjected to 2 cycles of homogenization (Qiagen TissueLyzer II) at 30Hz for 40 sec, rested for 5 min at 4 °C, and then subjected to 2 more cycles of homogenization. After homogenization, 750 µL of MTBE was added, and two cycles of extraction, again at 30Hz for 40 sec each with 5 min at 4 °C in between, were performed. Following the extraction, there was a final rest at 4 °C for 15 min. Samples were then centrifuged at 17,000g for 5 min at 4°C. 700 µL of the upper MTBE phase was transferred to a new 1.5-mL tube and evaporated to dryness using a speed-vac concentrator. Dried lipid samples were then reconstituted in 150 µL of isopropyl alcohol (IPA). After reconstitution in IPA, samples were again centrifuged at 17,000g for 15 min at 4°C. Concurrently with sample extractions, a process blank and a process blank spiked with an internal standard mix were prepared. During data collection, an aliquot of the NIST standard reference material SRM-1950 (https://tsapps.nist.gov/srmext/certificates/1950.pdf), Metabolites in Plasma, was analyzed along with samples and process blanks to evaluate end-to-end analysis consistency. All reconstituted extracts were stored at –80 °C before analysis.

b. Data acquisition. Samples were analyzed by UHPLC/MS and UHPLC/MS/MS in positive and negative ion modes. The UHPLC conditions were identical for all acquisitions, regardless of ionization polarity, dilution factor, or MS level (MS or MS/MS). The solvents consisted of A: 10 mM ammonium formate, 0.1% (v/v) formic acid, 60% (v/v) acetonitrile in water; and B: 10 mM ammonium formate, 0.1% (v/v) formic acid, 9% (v/v) acetonitrile, 1% (v/v) water in 2-propanol. The column was a Waters Acquity UPLC BEH C18 1.7µm 2.1mm x 50mm, with a guard column containing the same stationary phase with a 2.1mm x 5mm dimension. The gradient is shown in the table below.

Time	%A	%B	Flow
0.00 min	85.00 %	15.00 %	0.500 mL/min
2.40 min	70.00 %	30.00 %	0.500 mL/min
3.00 min	52.00 %	48.00 %	0.500 mL/min
13.20 min	18.00 %	82.00 %	0.500 mL/min
13.80 min	1.00 %	99.00 %	0.500 mL/min
15.40 min	1.00 %	99.00 %	0.500 mL/min
16.00 min	85.00 %	15.00 %	0.500 mL/min
20.00 min	85.00 %	15.00 %	0.500 mL/min

The column was maintained at 50 °C. Samples were placed in an autosampler and held at 8 °C until injection. The UHPLC was an Agilent model 1290 Infinity II with individual components consisting of a model G7120A binary pump, a model G7167B multi-sampler, a model G7116B column compartment, and a model G7110B isocratic pump. The HPLC was connected to the inlet port of an Agilent G6546A QTOF mass spectrometer, incorporating an Agilent JetStream dual ESI source. The column effluent was delivered to the sample nebulizer of the dual ESI source, and the isocratic pump delivered an internal calibrant to the reference nebulizer of the dual ESI source. QTOF parameters differed depending on the ionization polarity and MS level of the acquired data. Parameters for individual acquisition methods are

shown below.

Negative MS

Mass range	100-1500m/z
Scan rate	3 spectra/s
Gas temp	350°C
Gas flow	12 L/min
Nebulizer	25 psig
Sheath gas temp	350°C
Sheath gas flow	12 L/min
Capillary voltage	4750 V
Nozzle voltage	500 V
Fragmentor	170 V
Skimmer	50 V
Octopole RF peak	750 V
Reference masses	112.985587, 966.000725

Positive MS

Mass range	119-1500m/z
Scan rate	3 spectra/s
Gas temp	250°C
Gas flow	12 L/min
Nebulizer	35 psig
Sheath gas temp	300°C
Sheath gas flow	11 L/min
Capillary voltage	5000 V
Nozzle voltage	500 V
Fragmentor	190 V
Skimmer	75 V
Octopole RF peak	750 V
Reference masses	121.050873, 922.009798

Negative MS/MS

Mass range (MS)	100-1500m/z
Scan rate (MS)	3 spectra/s
Mass range (MS/MS)	40-1500 m/z
Scan rate (MS/MS)	3 spectra/s
Isolation width	Narrow
Collision energy	25.00 V
Gas temp	350°C
Gas flow	12 L/min
Nebulizer	25 psig
Sheath gas temp	375°C
Sheath gas flow	12 L/min
Capillary voltage	5000 V
Nozzle voltage	750 V
Fragmentor	190 V
Skimmer	45 V
Octopole RF peak	750 V
Reference masses	112.985587, 966.000725
Max precursors per cycle	2
Active exclusion	Yes
Active exclusion enabled after	1 spectrum
Active exclusion release after	0.10 min
Precursor exclusion	100-151m/z
Selected charges	1

Positive MS/MS

Mass range (MS)	119-1500m/z
Scan rate (MS)	3 spectra/s
Mass range (MS/MS)	40-1500 m/z
Scan rate (MS/MS)	3 spectra/s
Isolation width	Narrow
Collision energy	25.00 V
Gas temp	250°C
Gas flow	12 L/min
Nebulizer	35 psig
Sheath gas temp	300°C
Sheath gas flow	11 L/min
Capillary voltage	5000 V
Nozzle voltage	500 V
Fragmentor	190 V
Skimmer	75 V
Octopole RF peak	750 V
Reference masses	121.050873, 922.009798
Max precursors per cycle	2
Active exclusion	Yes
Active exclusion enabled after	1 spectrum
Active exclusion release after	0.10 min
Precursor exclusion	119-151m/z
Selected charges	1

c. Data analysis. Assignment of lipid identities to mass and retention time signal pairs were made using Lipid Annotator software (Agilent) [2] and the LC/MS/MS data. The Lipid Annotator uses the accurate mass of the precursor and product ions observed within the fragmentation spectra to assign a

dominant constituent lipid to a molecular feature in the data or, where that is not possible, a sum composition. Dominant compositions have lipid acyl chains and degree of unsaturation explicitly enumerated, whereas sum compositions only indicate the lipid class, total carbon number, and number of unsaturated sites. After the assignment of lipid identities by the Lipid Annotator, a database was exported containing the lipid, the mass, and the retention time. This database was then used by Profinder software (Agilent) to align retention times across samples and extract and integrate ion chromatograms for each lipid in each sample LC/MS data file.

SUPPLEMENTAL REFERENCES

1. Matyash, V.; Liebisch, G.; Kurzchalia, T. V.; Shevchenko, A.; Schwudke, D., Lipid extraction by methyl-tert-butyl ether for high-throughput lipidomics. *Journal of Lipid Research* **2008,** 49, (5), 1137-46.
2. Koelmel, J. P.; Li, X.; Stow, S. M.; Sartain, M. J.; Murali, A.; Kemperman, R.; Tsugawa, H.; Takahashi, M.; Vasiliou, V.; Bowden, J. A.; Yost, R. A.; Garrett, T. J.; Kitagawa, N., Lipid Annotator: Towards Accurate Annotation in Non-Targeted Liquid Chromatography High-Resolution Tandem Mass Spectrometry (LC-HRMS/MS) Lipidomics Using A Rapid and User-Friendly Software. *Metabolites* **2020,** 10, (3).

Primary antibodies used for immunostaining		
**Antibodies**	**Catalog #**	**Source**
Ki67	ab16667	Abcam, Cambridge, MA
cytokeratin 14 (K14)	MA5-11599	Abcam
keratin 10 (K10)	PRB-159P	BioLegend, San Diego, CA
keratin 6 (K6)	PRB-169P	BioLegend
loricrin (LOR)	pRB-145P	BioLegend
filaggrin (FLG)	pRB-417P	BioLegend
involucrin (IVL)	PRB-140C	BioLegend
Alexa Fluor® 594 anti-mouse CD11c	117346	BioLegend
Alexa Fluor® 594 anti-mouse CD4	100446	BioLegend
Ly-6G and Ly-6C (Gr-1)-biotin	553125	BD Pharmingen, San Diego, CA
Alexa Fluor® 594 anti-mouse CD8a	100758	BioLegend

Primary antibodies used for western blot analysis		
**Antibodies**	**Catalog #**	**Source**
RORα	sc28612	Santa Cruz Biotechnology, Santa Cruz, CA
keratin 10 (K10)	PRB-159P	BioLegend (Covance)
loricrin	pRB-145P	BioLegend (Covance)
filaggrin	pRB-417P	BioLegend (Covance)
involucrin	PRB-140C	BioLegend (Covance)
β-Actin (13E5)	4970S	Cell Signaling, Danvers, MA

Primer sequences of mouse genes for RT-PCR		
**Genes**	**Forward**	**Reverse**
Bmal1	GGCCAGAGCTTGTTTGACTA	GAAGTCCAGTCTTGGCATCA
Nr1d1	TACATTGGCTCTAGTGGCTCC	CAGTAGGTGATGGTGGGAAGTA
Krt16	GGTGGCCTCTAACAGTGATCT	TGCATACAGTATCTGCCTTTGG
Sprr2a2	GCCTTGTCGTCCTGTCATGT	GGCATTGCTCATAGCACACTAC
Krt10	GACAACTGACAATGCCAACG	GGTCAGCTCATCCAGTACCC
Ivl	AAAGCTTCAAGGGAACAGCA	CGGTTCTCCAATTCGTGTTT
Lor	GAGACAACAGAGCTGGAAGA	TCTGCAGAACAGGATACACC
Flg	GGAGGCATGGTGGAACTGA	TGTTTATCTTTTCCCTCACTTCTACATC
Hrnr	GGCTCCCAAGAATATAGCTCTGG	GATGTCTGCCTGTTGACTGTC
Krt77	CCAGGTGCTACAGACAAAATGG	GCTGACTGATGAACTCCTCGAA
Asah1	TGTTACTGGGATTCCTCTAGGAG	CTTCTGTGACAACCCAAGTGT
Cat	TGGCACACTTTGACAGAGAGC	CCTTTGCCTTGGAGTATCTGG
18S	AGG TTC TGG CCA ACG GTC TAG	CCC TCT ATG GGC TCG AAT TTT
